# Enhanced Broad Spectrum In Vitro Antiviral Efficacy of 3-F-4-MeO-Bn, 3-CN, and 4-CN Derivatives of Lipid Remdesivir Nucleoside Monophosphate Prodrugs

**DOI:** 10.1016/j.antiviral.2023.105718

**Published:** 2023-09-25

**Authors:** Rachel E. McMillan, Michael K. Lo, Xing-Quan Zhang, James R. Beadle, Nadejda Valiaeva, Aaron F. Garretson, Alex E. Clark, Jon Freshman, Joyce Murphy, Joel M. Montgomery, Christina F. Spiropoulou, Robert T. Schooley, Karl Y. Hostetler, Aaron F. Carlin

**Affiliations:** 1 Division of Infectious Diseases and Global Public Health, Department of Medicine, University of California San Diego, School of Medicine, La Jolla, California, USA.; 2 Department of Pathology, University of California San Diego, School of Medicine, La Jolla, California, USA.; 3 Viral Special Pathogens Branch, Centers for Disease Control and Prevention, Department of Health and Human Services, Atlanta, Georgia, USA.

**Keywords:** RNA virus, lipid prodrugs, remdesivir, remdesivir nucleoside, GS-441524, GS-5734, V2043, broad spectrum antiviral, flavivirus, Zika virus, Dengue virus, filovirus, Ebola virus, paramyxovirus, henipavirus, Nipah virus, Hendra virus, pneumovirus, respiratory syncytial virus, human coronavirus 229E, antiviral agents, respiratory viruses, hemorrhagic fever viruses

## Abstract

Broad spectrum oral antivirals are urgently needed for the early treatment of many RNA viruses of clinical concern. We previously described the synthesis of 1-O-octadecyl-2-O-benzyl-glycero-3-phospho-RVn (V2043), an orally bioavailable lipid prodrug of remdesivir nucleoside (RVn, GS-441524) with broad spectrum antiviral activity against viruses with pandemic potential. Here we compared the relative activity of V2043 with new RVn lipid prodrugs containing sn-1 alkyl ether or sn-2 glycerol modifications. We found that 3-F-4-MeO-Bn, 3-CN-Bn, and 4-CN-Bn sn-2 glycerol modifications improved antiviral activity compared to V2043 when tested in vitro against clinically important RNA viruses from 5 virus families. These results support the continued development of V2043 and sn-2 glycerol modified RVn lipid prodrugs for the treatment of a broad range of RNA viruses for which there are limited therapies.

## 1. Introduction

Remdesivir (RDV; GS-5734) is an adenosine analog prodrug that binds and inhibits viral RNA-dependent RNA polymerase and exhibits broad-spectrum antiviral activity in vitro and in vivo. When administered to nonhospitalized COVID-19 infected patients, RDV reduced hospitalization or death by 87% in individuals at high risk for progression to severe disease (Gottlieb et al. 2022).

RDV requires intravenous administration, limiting its utility for outpatient treatment, is rapidly metabolized in the blood, and is dose-limited by liver toxicity (Antinori et al. 2020, Carothers et al. 2020, Goldman et al. 2020, Humeniuk et al. 2020, Tempestilli et al. 2020). To overcome these limitations, we previously generated orally available lipid prodrugs of remdesivir nucleoside (RVn) that resemble lysophospholipids, which are normally absorbed intact from the gastrointestinal tract (Schooley et al. 2021). Of these analogs, 1-O-octadecyl-2-O-benzyl-glycero-3-phospho-RVn (V2043) was orally bioavailable, stable in plasma, and less toxic than RDV in human liver cells. V2043 demonstrated potent antiviral activity against SARS-CoV-2 and other RNA viruses in vitro and reduced viral loads in the lungs of mice infected with SARS-CoV-2 when administered orally (Carlin et al. 2023, Lo et al. 2021, Schooley et al. 2021). We also found that 3-F-4-MeO-Bn, 3-CN-Bn, and 4-CN-Bn modifications at the sn-2 glycerol enhanced antiviral activity against SARS-CoV-2 (Carlin et al. 2023).

Here, we investigate the in vitro antiviral activity of V2043 and various analogs, including 3-F-4-MeO-Bn, 3-CN-Bn, or 4-CN-Bn sn-2 glycerol modifications, against members of the *Flaviviridae*, *Filoviridae*, *Pneumoviridae*, *Paramyxoviridae*, and *Coronaviridae* families. We show that 3-F-4-MeO-Bn, 3-CN-Bn, and 4-CN-Bn sn-2 glycerol modifications improve the antiviral efficacy of RVn lipid prodrugs against most RNA viruses tested. Collectively, this work demonstrates the broad-spectrum in vitro activity of RVn lipid prodrugs and identifies specific sn-2 glycerol modifications that improve broad spectrum antiviral potency.

## 2. Materials and methods

### 2.1 Compounds

Compounds were synthesized at the University of California, San Diego, Nanosyn, Inc. (Santa Clara, CA and J-Star Research, South Plainfield, NJ. Detailed synthesis methods and characterization are provided in Carlin et al. JMedChem 2023.

### 2.2 Cell culture

Huh7.5 cells were acquired from APATH, LLC and grown in DMEM (Invitrogen) with 10% FBS and 1% penicillin/streptomycin (Gibco). Human telomerase reverse-transcriptase (hTERT) immortalized cell lines were cultured and maintained as previously described (Lo et al. 2020, Lo et al. 2021, Siegel et al. 2017). Briefly, microvascular endothelial (TIME) (CRL-4025, ATCC) cells were grown in vascular cell basal endothelial growth media (PCS-100-030, ATCC) supplemented with microvascular endothelial growth kit-VEGF (PCS-110-041, ATCC). hTERT-immortalized human small airway epithelial cells (HSAEC1-KT) (CRL-4050, ATCC) were cultured in epithelial cell basal medium (PCS-300-030, ATCC) and supplemented with Bronchial epithelial cell growth kit (PCS-300-40, ATCC). All cell lines were cultured at 37° C, 5% CO2. HeLa cells were acquired from ATCC and grown in DMEM (Gibco) with 10% FBS, 100 units/ml penicillin, and 100 mcg/ml streptomycin. HEp-2 and MRC-5 cells were acquired from ATCC and grown in EMEM (Gibco) with 10% FBS, 100 units/ml penicillin, and 100 mcg/ml streptomycin.

### 2.3 Differentiation of monocyte derived dendritic cell (moDCs) from primary human monocytes

Blood was drawn from healthy human donors under IRB #181624. Blood on top of Histopaque was centrifuged at 400g for 45 minutes at 4 ºC without acceleration. The buffy coat was isolated and washed with PBS containing 0.02% w/v EDTA. To lyse remaining red blood cells, the cell pellet was resuspended in molecular grade water. Subsequently, 10X PBS was added to a final concentration of 1X PBS prior to filtering cells with a 70 μm filter. Cells were centrifuged at 300 x g for 10 min at 4 ºC and washed once with PBS containing 0.02% w/v EDTA. Monocytes were isolated following the Pan Monocyte Isolation Kit (Miltenyi Biotec) instructions. Cells were plated at a seeding density ranging from 1.0 × 10^6^ to 1.7 × 10^6^ cells/mL in 6 well plates in complete DC media. Complete DC media consists of RPMI 1640 (Gibco) containing 10% FBS, 1% penicillin/streptomycin, 1% HEPES, 100 ng/mL recombinant human granulocyte-macrophage colony stimulating factor, and 100 ng/mL recombinant human interleukin 4. Cells were incubated for a total of 9 days at 37 ºC; media was changed on day 4 and 8, and cell differentiation was checked on day 7 or 8. Compounds and virus were added on day 8.

### 2.4 Drug treatments

For antiviral assays, compounds were added to cells at 0.013, 0.041, 0.123, 0.371, 1.11, 3.33, 10, and 30 μM immediately prior to addition of virus and were present for the duration of infection. For cell toxicity assays, compounds were added to cells at 1.23, 3.70, 11.11, 33.33, and 100 μM. moDCs and Huh7.5 cells were incubated with compounds or controls for 24 and 48 hours, respectively. For antiviral assays in TIME and HSAEC1-KT cells, compounds were added to cells at 0.0023, 0.0068, 0.021, 0.062, 0.19, 0.56, 1.67, and 5 uM 1 h prior to addition of virus and were present for the duration of infection. For RSV antiviral assays, compounds were added to cells at 0.0025, 0.01, 0.04, 0.16, 0.64, and 2.5 μM immediately prior to addition of virus and were present for the duration of infection. For HCoV 229E antiviral assays, compounds were added to cells at 0.0025, 0.01, 0.04, 0.16, 0.64, 2.5, and 10 μM immediately prior to addition of virus and were present for the duration of infection. For HeLa, HEp-2 and MRC-5 cell toxicity assays, compounds were added to cells at 0.0125, 0.05, 0.20, 0.80, 3.20, 12.5 and 50 μM. HeLa, HEp-2, and MRC-5 cells were incubated with compounds or controls for 5–7 days, respectively.

### 2.5 Viral infection

Huh7.5 cells were seeded at 18,000 cells per well in flat bottom 96 well plates 24 hours prior to treatment. Cells were treated with compounds or controls at the indicated concentrations. Following drug treatment, Huh7.5 cells were infected with DENV2 UIS 353 at an MOI of 0.02 or ZIKV PRVABC59 at an MOI of 0.0110 – 0.0125. Compounds and virus were incubated on cells for 48 hours. On day 8 of moDC differentiation, cells were seeded at 50,000 cells per well in 96 well round bottom plates. After compound or control treatment at the indicated concentrations, moDCs were infected with DENV2 UIS 353 at an MOI of 0.33 or ZIKV PRVABC59 at an MOI of 1 for 24 hours. HeLa and HEp-2 cells were seeded at 10^5^ cells per well and MRC-5 cells were seeded at 10^4^ cells per well, respectively, in flat bottom 96 well plates 24 hours prior to treatment. Cells were treated with compounds or controls at the indicated concentrations. Following drug treatment, HeLa, HEp-2, and MRC-5 cells were infected with RSV at an MOI of 0.01 or HCoV 229E at an MOI of 0.01.

### 2.6 Viruses

ZIKV PRVABC59 and DENV2 UIS 353 were acquired from the World Reference Center for Emerging Viruses and Arboviruses. ZIKV PRVABC59 and DENV2 UIS 353 were expanded on C6/36 Aedes albopictus mosquito cells. Virus was titrated on 70–90% confluent U2OS cells. U2OS cells were seeded in a 6 well plate at 300,000 to 400,000 cells per well, or in a 12 well plate at 100,000 cells per well and incubated overnight. 5-fold serial dilutions of virus were diluted in U2OS media with 2% FBS and incubated on the cells for one hour, rocking plates every 15 minutes. Cells were washed once with PBS and complete media was added to cells. After 24 hours, cells were prepared for flow cytometry according to the BD Fixation/Permeabilization kit. Virus was stained using AF647-conjugated 4G2 mAb. Infection rate was determined following flow cytometry using the ACEA Novocyte flow cytometer and subsequent FlowJo analysis. The henipaviruses used in this study (NiV-B, rNiV-ZsG (NiV-M genotype), and HeV (Chua et al. 2000, Harcourt et al. 2004, Murray et al. 1995) were propagated and titered on Vero (ATCC, CCL-81) cells. The recombinant ebolavirus used in this study (Albariño et al. 2015) was propagated and titered on Huh7 cells (APATH, LLC). Prior to conducting antiviral assays, all viruses were titered on both TIME and HSAEC1-KT cells to preclude differences in cell type-specific infectivity.

### 2.7 Cell viability assay

Huh7.5 cells were plated at 10,000 cells per well in opaque 96 well plates and incubated overnight. Cells were treated at the indicated concentrations with compounds or controls for 48 hours. On day 8 of differentiation, moDCs were seeded at 50,000 cells per well in opaque 96 well plates and treated with compounds or controls at the indicated concentrations for 24 hours. HeLa and HEp-2 were plated at 10^5^ cells per well and MRC-5 cells were plated at 10^4^ cells per well in opaque 96 well plates and incubated overnight. Cells were treated at the indicated concentrations with compounds or controls for 5–7 days. Cell Titer Glo or Cell Titer Glo 2.0 reagent (Promega) were used according to the manufacturer’s instructions to measure cell toxicity by ATP levels. Luminescence was recorded with the Veritas Microplate Luminometer (Turner BioSystems) or HD1 Synergy plate reader. CC_50_ values were calculated using Prism 9 by normalizing cell viability to the DMSO controls.

### 2.8 Immunofluorescence of infected Huh7.5s

Huh7.5 cells were washed three times with PBS and fixed in 4% paraformaldehyde for 30 minutes at room temperature (RT). Cells were washed three times with PBS and permeabilized with 0.1% Triton X in a solution of 1% BSA for 30 minutes at RT. Cells were incubated with pan flavivirus mouse antibody, 4G2 in 1% BSA with 0.1% Triton X overnight at 4C. After three PBS washes, cells were stained Alexa fluor 594 goat anti-mouse IgG2a(y2a) (Invitrogen) and SYTOX Green nucleic acid stain (Invitrogen) for 1 hour at RT. Cells were washed three times with PBS and imaged on the Incucyte S3 System (Sartorius). IC50 values were calculated using Prism 9 by normalizing infection rate (cells with AF594 signal/cells with Sytox Green signal) to the DMSO controls.

### 2.9 Flow cytometry of infected moDCs

moDCs were centrifuged for 10 minutes at 200g and washed once with PBS. Cells were stained with Zombie Violet Fixable Viability stain according to the kit instructions and incubated for 15 minutes at RT. moDCs were fixed and permeabilized according to the BD Fixation/Permeabilization kit, using AF647-conjugated 4G2 mAb to stain for ZIKV or DENV viral envelope protein. Infection rate was determined following flow cytometry using the Novocyte flow cytometer and FlowJo analysis.

### 2.10 Fluorescence reporter-based assays (REP)

Recombinant NiV (rNiV-ZsG) (Lo et al. 2014) and EBOV (rEBOV-ZsG) (Albariño et al. 2015) expressing ZsGreen1 fluorescent protein (ZsG) were assayed for total fluorescence intensity by using an H1 Synergy plate reader (Biotek) as previously described (Lo et al. 2021). HSAEC1-KT and TIME cells were seeded at 1–2 × 10^4^ cells per well in black opaque side clear bottom 96-well plates (Corning 3603, Corning, NY) and compounds were added to the assay plates for 1 h. Assay plates were transferred to the BSL-4 suite and infected with 0.25 TCID_50_ per cell of the respective virus and were read at 72 hours post-infection (hpi). Fluorescence signal intensity assayed in DMSO-treated, virus-infected cells were set as 100% ZsGreen fluorescence. Data points and error bars for all reporter assays indicate the mean value and standard deviation of 3 biological replicates and are representative of at least 3 independent experiments for every compound tested. Concentrations of compound that inhibited 50% of the green fluorescence signal (EC_50_) were calculated from dose response data fitted to the mean value of experiments performed for each concentration in the 8-point, 3-fold dilution series using a 4-parameter non-linear logistic regression curve with variable slope using GraphPad Prism 9 (GraphPad Software, La Jolla, CA, USA).

### 2.11 Cytopathic effect (CPE) assay

CPE inhibition assays were conducted as previously described (Lo et al. 2020, Lo et al. 2021). HSAEC1-KT and TIME cells were seeded at 1–2 × 104 cells per well in white opaque 96-well plates, and compounds were added to the assay plates. Assay plates were transferred to the BSL-4 suite as per biocontainment requirements, infected with 0.01–0.5 TCID_50_ per cell, and were analyzed with CellTiter-Glo 2.0 (Promega, Madison, WI) at 72 hpi in a HD1 Synergy plate reader. Values were normalized to uninfected cell controls according to % viability as follows: % viability = [(specific value-reference value)/(DMSO control value – reference value)] × 100. Reference values were derived from control wells without cells. Uninfected cell control values (after subtraction of reference values) were set at 100% inhibition of CPE. EC50 values were calculated using four-parameter variable slope non-linear regression fitting of values.

### 2.12 Statistical analysis

All statistical analyses were performed using Prism 9.3.1 (GraphPad Software). To calculate CC_50_ and EC_50_ values for each compound, curves were fit using [inhibitor] vs. normalized response (variable slope). To compare IC50 values of V2043 versus V2051-V2055, data was log transformed and a two-tailed unpaired t test was performed. To compare IC50 values of V2043 with V2067 and V2067, an ordinary one-way ANOVA that assumed Gaussian distribution of residuals and equal standard deviations was performed on log transformed data; this test compared the mean of each compound to that of V2043. The IC90 values of each compound were calculated using the IC50 and Hillslope values calculated in prism, IC90 = IC50(1/9^(Hillslope)^). Data is presented as the mean ± standard deviation of at least three independent experiments. Data from Huh7.5 cells was generated from at least 3 experiments. Experiments with moDCs were generated from at least 3 different donors. A “p” value of less than 0.05 was considered significant.

## 3. Results

### 3.1 Modification of R1 and R2 groups of V2043

**Figure 1:**
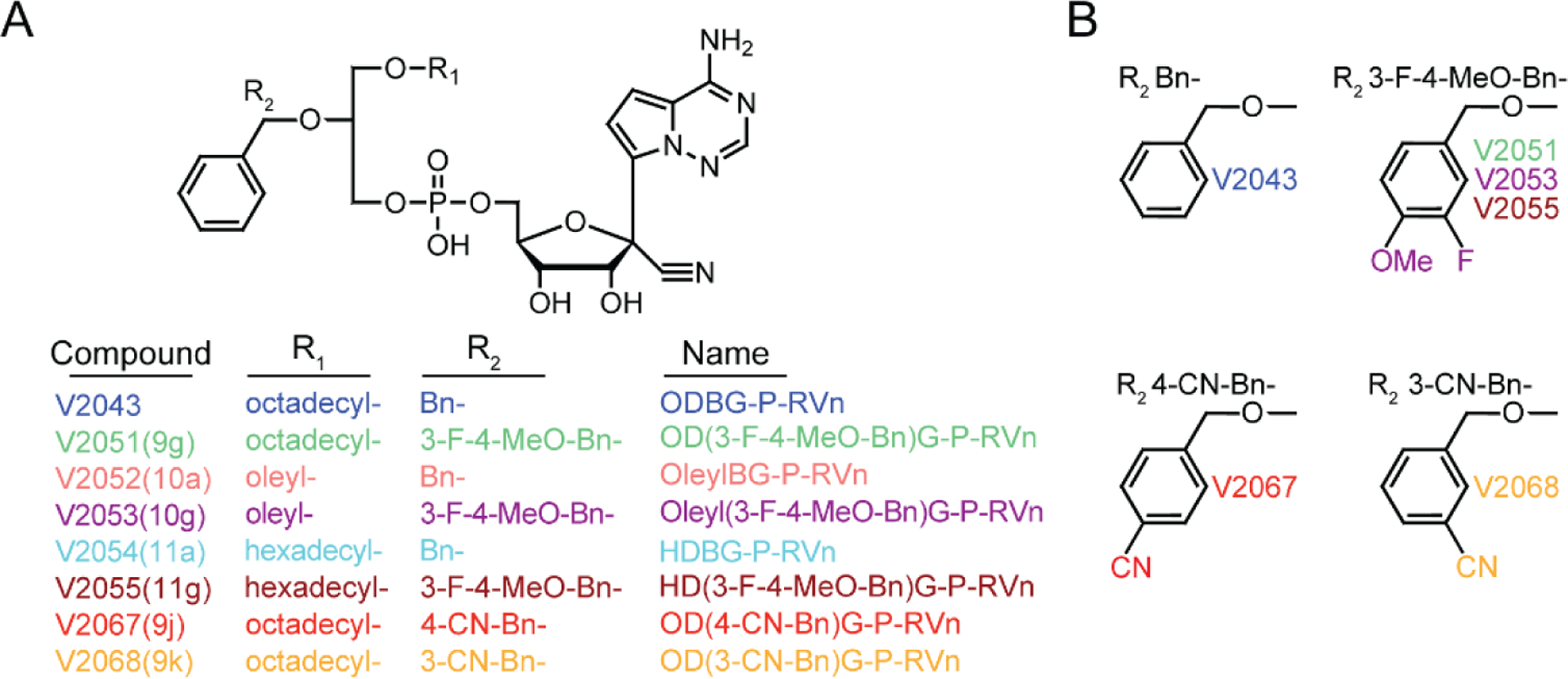
Structures of V2043 and RVn lipid prodrugs containing sn-1 alkyl ether or sn-2 glycerol modifications. (A) V2043 structure and R1 and R2 modifications of RVn lipid prodrug analogues. (B) Structures of R2 modifications.

We previously generated analogs of V2043 by modifying the R1 and/or R2 groups (Fig 1A and 1B) (Carlin et al. 2023). At R1, compounds V2043, V2051, V2067, V2068 have an octadecyl group, V2052 and V2053 have an oleyl group, and V2054 and V2055 have a hexadecyl group. The R2 benzyl group present in V2043 was modified with a 3-F-4-MeO-Bn for compounds V2051, V2053, and V2055 or a cyano group at the 4’, or 3’ position for compounds V2067 and V2068, respectively.

### 3.2 Addition of 3-CN, 4-CN or 3-F-4-MeO substituents to the benzyl group enhances antiviral activity against Dengue virus and Zika virus infection in vitro

In humans, flaviviruses initially replicate in innate immune cells, such as dendritic cells, and subsequently spread hematogenously leading to hepatocyte infection (Pόvoa et al. 2014, Schmid et al. 2014, Win et al. 2019). We tested the ability of V2043, V2051, V2052, V2053, V2054, V2055, V2067, and V2068 to inhibit replication of dengue virus (DENV) and Zika virus (ZIKV) in a human hepatocyte cell line, Huh7.5, and primary human monocyte-derived dendritic cells (moDCs). moDCs and Huh7.5 cells were treated with compound concentrations ranging from 0.0137 to 30 μM and infected with DENV and ZIKV. To maximize infection, Huh7.5 cells were infected for 48 hours and moDCs were infected for 24 hours (Branche et al. 2022). Infection rate was determined by immunofluorescence for infected Huh7.5 cells and flow cytometry for infected moDCs. For both immunofluorescence and flow cytometry, changes in the percentage of cells positive for intracellular viral antigen were quantified.

**Figure 2:**
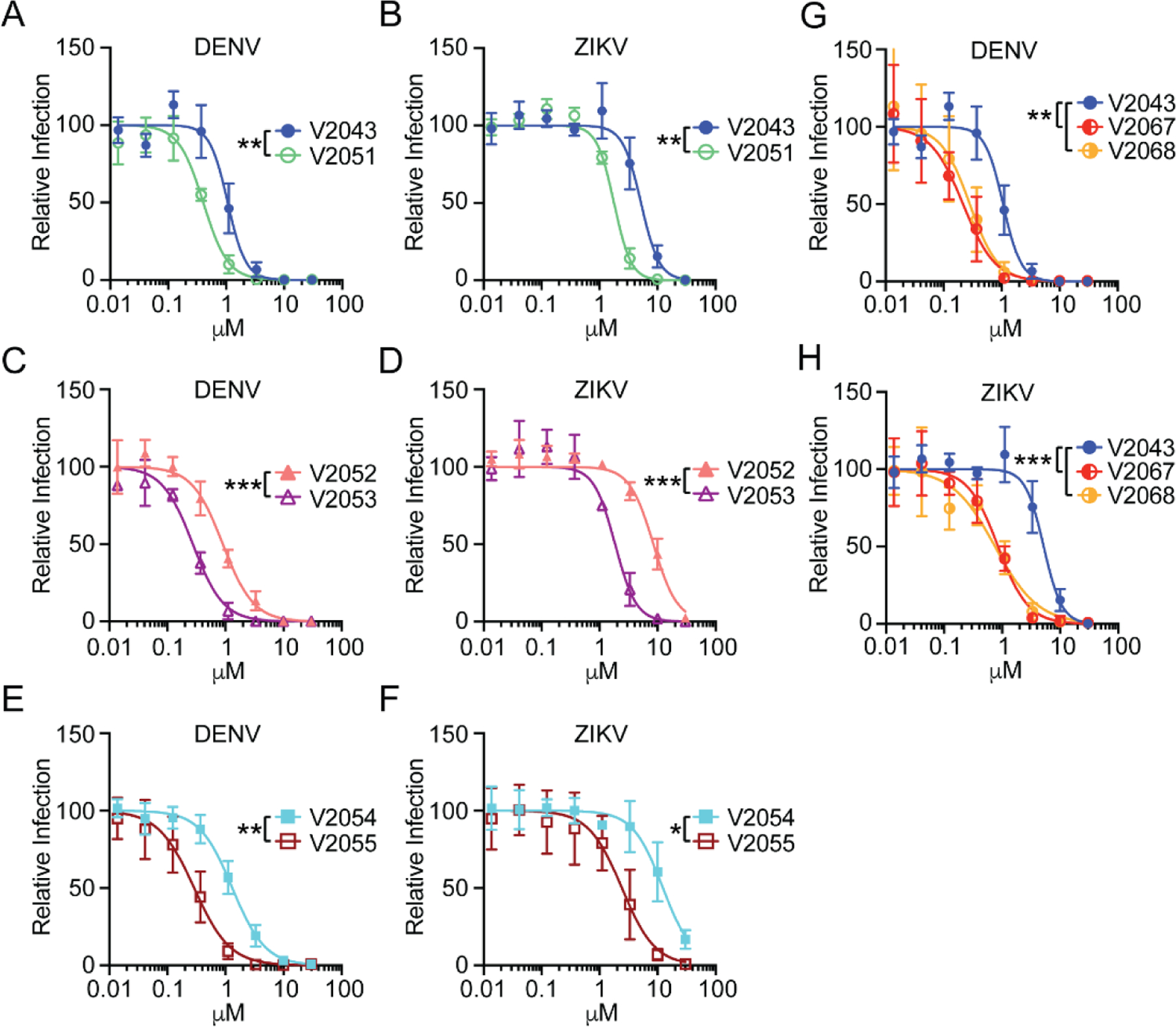
3-F-4-MeO-Bn, 3-CN, and 4-CN R_2_ substituted compounds have increased antiviral activity against DENV and ZIKV in Huh7.5, a human hepatocyte cell line. (A-F) Antiviral dose-response curves comparing compounds that differ solely by a 3-F-4-MeO-Bn modification (A and B V2043 versus V2051), (C and D V2052 versus V2053), and (E and F V2054 versus V2055) during (A, C, E) DENV and (B, D, F) ZIKV infection of Huh7.5 cells. Antiviral dose-response curves comparing compounds that differ solely by a 3-CN-Bn or 4-CN-Bn compared to V2043 in (G) DENV or (H) ZIKV infection of Huh7.5 cells. Immunofluorescence was used to quantify changes in intracellular viral antigen, detected by staining for pan-flavivirus envelope epitope, 4G2. Data shown are generated from at least 3 independent experiments performed in duplicate. Error bars represent standard deviation. Log_10_EC_50_ values from each experiment were compared by unpaired t test (A-F) or compared to V2043 by one-way ANOVA (G and H) with Dunnett’s correction for multiple comparisons * P < 0.05, ** P < 0.01, ***P < 0.001.

**Figure 3:**
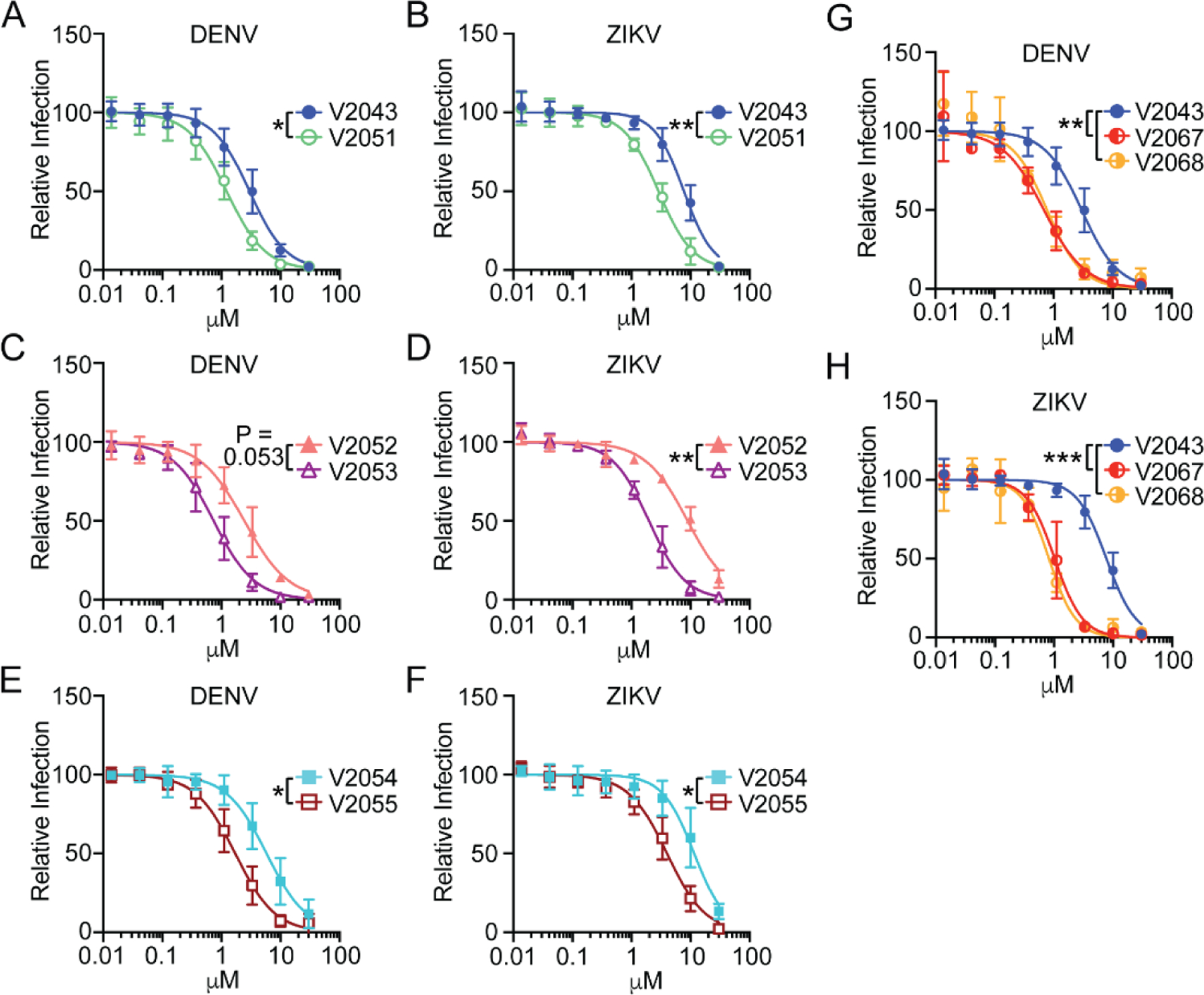
3-F-4-MeO-Bn, 3-CN, and 4-CN R_2_ substituted compounds have increased antiviral activity against DENV and ZIKV in primary human monocyte-derived dendritic cells (moDC). (A-H) Antiviral dose-response curves for indicated compounds during (A, C, E, G) DENV and (B, D, F, H) ZIKV infection of moDCs. Flow cytometry was used to quantify changes in intracellular viral antigen, detected by staining for pan-flavivirus envelope epitope, 4G2. (I-J) Data shown are generated from at least 3 independent experiments performed in duplicate. Error bars represent standard deviation. Log_10_EC_50_ values from each experiment were compared by unpaired t test (A-F) or compared to V2043 by one-way ANOVA (G and H) with Dunnett’s correction for multiple comparisons * P < 0.05, ** P < 0.01, ***P < 0.001.

All compounds exhibited dose-dependent inhibition of DENV and ZIKV in Huh7.5s and moDCs (Fig 2A–2F, Fig 3A–3F). Compared to related analogs with an unmodified benzyl R2 group, V2051, V2053, and V2055 have significantly lower 50% inhibitory effective concentration (EC_50_) values in ZIKV -infected Huh7.5 cells and moDCs; V2051 and V2055 also have significantly lower EC_50_ values in DENV -infected moDCs and Huh7.5 cells (Fig 2A–2F, Fig 3A–3F, Tables 1 and 2). V2053 had improved antiviral efficacy compared to V2052 in DENV -infected Huh7.5 cells and moDCs, although the difference in moDCs did not reach statistical significance (*P* = 0.053) (Fig 2C, 3C, Tables 1 and 2). Relative to V2043, all 3-F-4-MeO R2 modified compounds have higher selective indices (SI) in both cell types (Tables 1 and 2). These data suggest that in DENV and ZIKV -infected Huh7.5 cells and moDCs, addition of a 3-F-4-MeO to the R2 benzyl group increases the antiviral activity of V2043, irrespective of R1 side chain length.

To determine if the addition of a CN group to the R2 benzyl of V2043 increases antiviral activity, we compared the relative activity of V2067 and V2068 to inhibit viral replication in DENV and ZIKV infected Huh7.5 cells and moDCs. We found that V2067 and V2068 induce a dose-dependent inhibition of DENV and ZIKV replication in both cell types (Fig 2G and 2H, Fig 3G and 3H). Compared to V2043, V2067 and V2068 have significantly lower EC_50_ values in DENV and ZIKV -infected Huh7.5 cells and moDCs (Fig 2G and 2H, Fig 3G and 3H, Tables 1 and 2). Additionally, in both DENV and ZIKV -infected moDCs and Huh7.5 cells, the SI of V2067 and V2068 exceeds that of V2043. Altogether, this data suggests that both 3-F-4-MeO and - CN R2 substitutions increase antiviral activity against DENV and ZIKV in Huh7.5 cells and moDCs.

### 3.3 V2043 and modified analogs exhibit broad spectrum antiviral efficacy

To establish if 3-F-4-MeO and -CN R2 modified compounds exhibit antiviral efficacy against additional RNA viruses of medical importance, we tested the ability of these compounds to inhibit viruses in the *Filoviridae*, *Paramyxoviridae, Pneumoviridae*, and *Coronaviridae* families in vitro.

**Figure 4:**
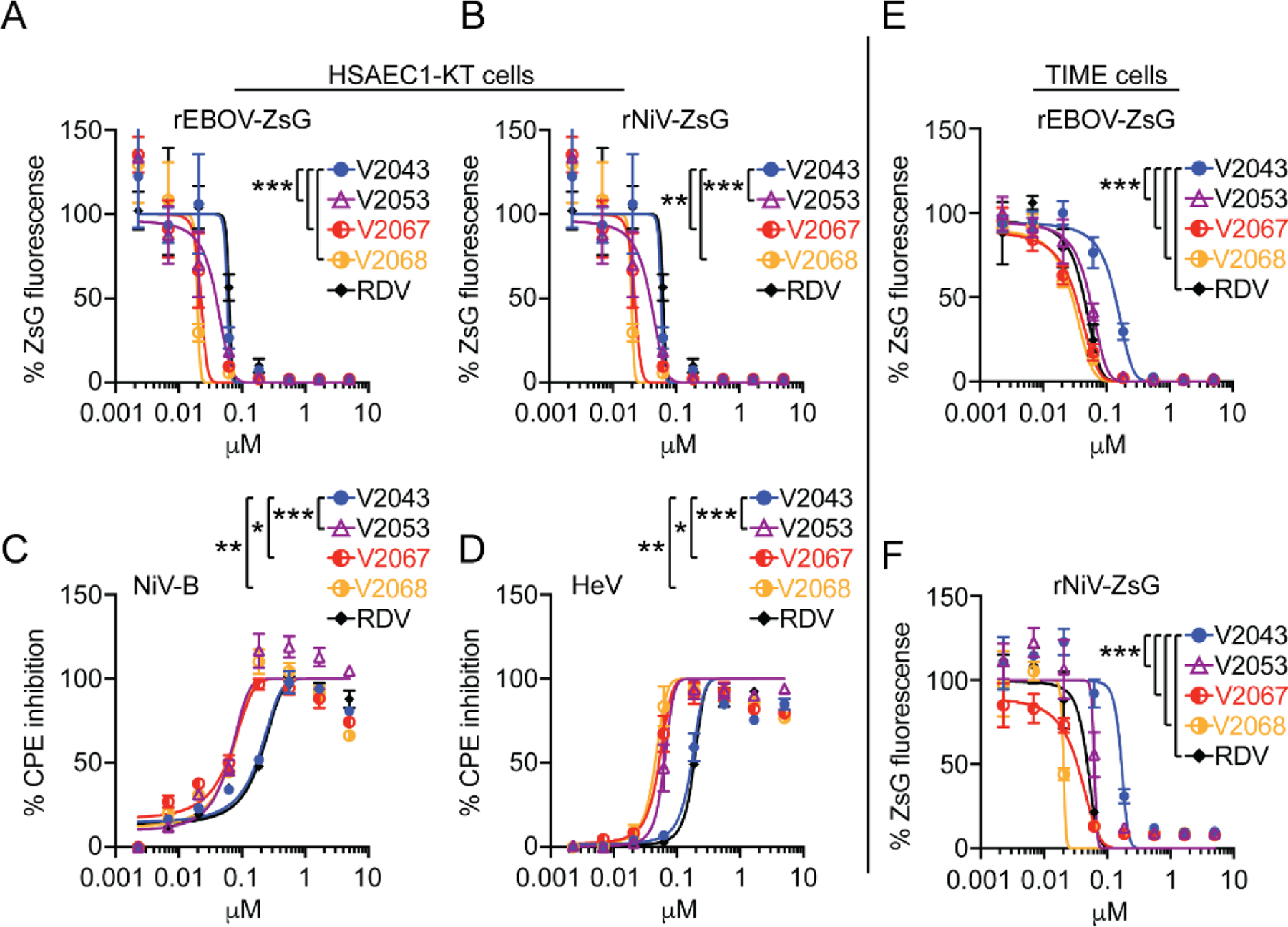
3-F-4-MeO-Bn, 3-CN, and 4-CN R_2_ substituted compounds have increased antiviral activity over V2043 against EBOV, NiV, and HeV. Antiviral dose-response curves for indicated compounds in in either HSAEC1-KT or TIME cells infected with recombinant reporter rEBOV-ZsG (A, E,) and rNiV-ZsG (B, F) viruses or with wild-type NiV-B (C) and HeV (D). Cells infected with recombinant viruses expressing ZsGreen1 protein (ZsG) were assayed for levels of fluorescence normalized to levels observed from DMSO-treated virus infected controls. HSAEC1-KT cells infected with wild-type NiV-B and HeV were assayed for cytopathic effect (CPE) based on cellular ATP levels using CellTiterGlo 2.0. CPE was normalized to cellular ATP levels observed in DMSO-treated uninfected controls. Data shown are representative at least 3 independent experiments performed in triplicate. Error bars represent standard deviation. LogEC_50_ values from each experiment were compared to V2043 by one-way ANOVA with Dunnett’s correction for multiple comparisons * P < 0.05, ** P < 0.01, ***P < 0.001.

Having previously established the activity of V2043 against multiple filoviruses and paramyxoviruses (Lo et al. 2021), we compared the antiviral activities of V2043, V2051, V2053, V2055, V2067, V2068, and RDV against a subset of these families including Ebola (rEBOV-ZsG), Nipah (rNiV-ZsG, NiV-B), and Hendra (HeV) viruses in primary-like hTERT-immortalized human dermal microvascular endothelial (TIME) and human small airway epithelial (HSAEC1-KT) cells in vitro (Table 3 and 4). Antiviral efficacy was determined either by measuring levels of ZsGreen1 protein (ZsG) fluorescence emission (Fig 4A, 4B, 4E, 4F), or by measuring cytopathic effect (CPE) based on cellular ATP levels (Fig 4C and 4D). All 7 compounds exhibited dose-dependent inhibition against all viruses tested. In rEBOV-ZsG infected HSAEC1-KT cells, V2043 and RDV had similar EC50 values, whereas EC50 values for V2051, V2053, V2055, V2067 and V2068 were consistently 3 to 4-fold lower in comparison (Table 3). In rNiV-ZsG infected HSAEC1-KT cells, V2051 had 2 to 3-fold lower EC50 values than V2043 and RDV, while V2053, V2055, V2067, and V2068 had 4 to 5-fold lower EC50 values. In HSAEC1-KT cells infected with wild-type NiV-B, V2053, V2055, V2067, and V2068 had 2 to 3-fold lower EC50 values than V2043 and RDV. In HeV infected HSAEC1-KT cells, V2053, V2055, and V2068 had 4-fold lower EC50 values than V2043 and RDV, while V2051 and V2067 only showed 2-fold lower EC50 values. In TIME cells infected with reporter rEBOV-ZsG, V2051, V2053, and V2055 had approximately 3-fold lower EC50 values than V2043, while V2067, V2068, and RDV had at least 5-fold lower EC50 values. In rNiV-ZsG infected TIME cells RDV, V2053, and V2055 have 3 to 4-fold lower EC50 values than V2043, while V2067 and V2068 have at least 5-fold lower EC50 values (Table 4). Supporting the antiviral efficacy of 3-F-4-MeO and -CN R2 modified compounds, the SI of V2051, V2053, V2055, V2067, and V2068 exceed that of V2043 in both HSAEC1-KT and TIME cells, suggesting that these compounds have significantly higher antiviral efficacy than V2043 against Ebola, Nipah and Hendra viruses.

**Figure 5:**
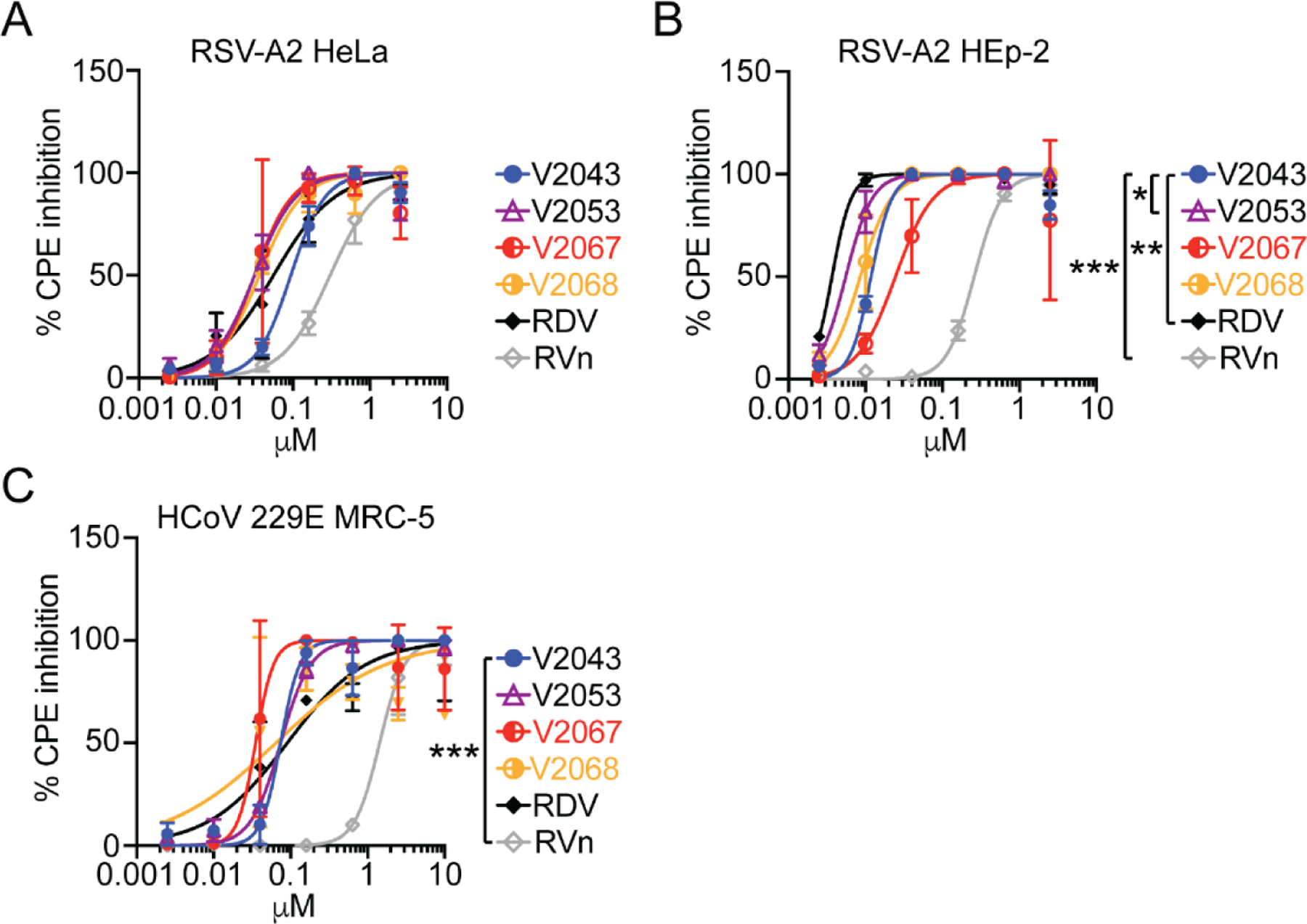
Antiviral activity of RDV, V2043, and 3-F-4-MeO-Bn and CN R_2_ substituted compounds against RSV and human coronavirus 229E. Antiviral dose-response curves for indicated compounds during (A-B) RSV and (C) HCoV 299E infection of indicated cell types. Data shown are generated from at least 3 independent experiments performed in duplicate. Error bars represent standard deviation. Log_10_EC_50_ values from each experiment were compared to V2043 by one-way ANOVA with Dunnett’s correction for multiple comparisons * P < 0.05, ** P < 0.01, ***P < 0.001.

We then evaluated the antiviral activity of V2043, V2051, V2053, V2055, V2067, and V2068 against respiratory syncytial virus (RSV)-A2 in HeLa and HEp-2 cells and human coronavirus-229E (HCoV-229E) in MRC-5 cells (Table 4 and 5). Antiviral efficacy was determined by measuring inhibition of cytopathic effect, and significance testing was performed on -CN R2 substituted compounds and our top 3-F-4-MeO R2 substituted compound, V2053 (Fig 5A–5C). RDV, RVn, V2043, V2053, V2067, and V2068 exhibit dose-dependent inhibition of RSV-A2 and HCoV-2 229E in vitro. In RSV -infected HeLa cells, V2053, V2067, and V2068 have a lower EC_50_ than V2043, although these differences are not significant (Fig 5A, Table 5). Supporting the antiviral efficacy of 3-F-4-MeO and -CN R2 modified compounds, the SI of V2051, V2053, V2055, and V2068 exceed that of V2043. V2067 has a lower SI value than V2043, which can be explained by its cytotoxicity in HeLa cells (Supp Fig 1). In RSV -infected HEp-2 cells, V2053 had significantly lower EC_50_ values than V2043 (Fig 5B, Table 5). Although there is not a significant difference between the EC_50_ values of V2043 and V2067-V2068, the SI of V2067 and V2068 exceed that of V2043. Overall, this suggests that 3-F-4-MeO modified V2053 has significantly higher antiviral efficacy than V2043 against RSV in HEp-2 cells.

When testing these compounds against HCoV-229E in MRC-5 cells, we found that -CN modified compounds, V2067 and V2068, have lower EC_50_ values than V2043 (Fig 5C, Table 5). The SI of 3-F-4-MeO compounds, V2051, V2053, and V2055 do not exceed that of V2043 (Table 5). However, V2067 and V2068 have higher SIs than V2043. This suggests that -CN but not 3-F-4-MeO modifications improve antiviral activity against HCoV-229E in MRC-5 cells.

**Figure 6:**
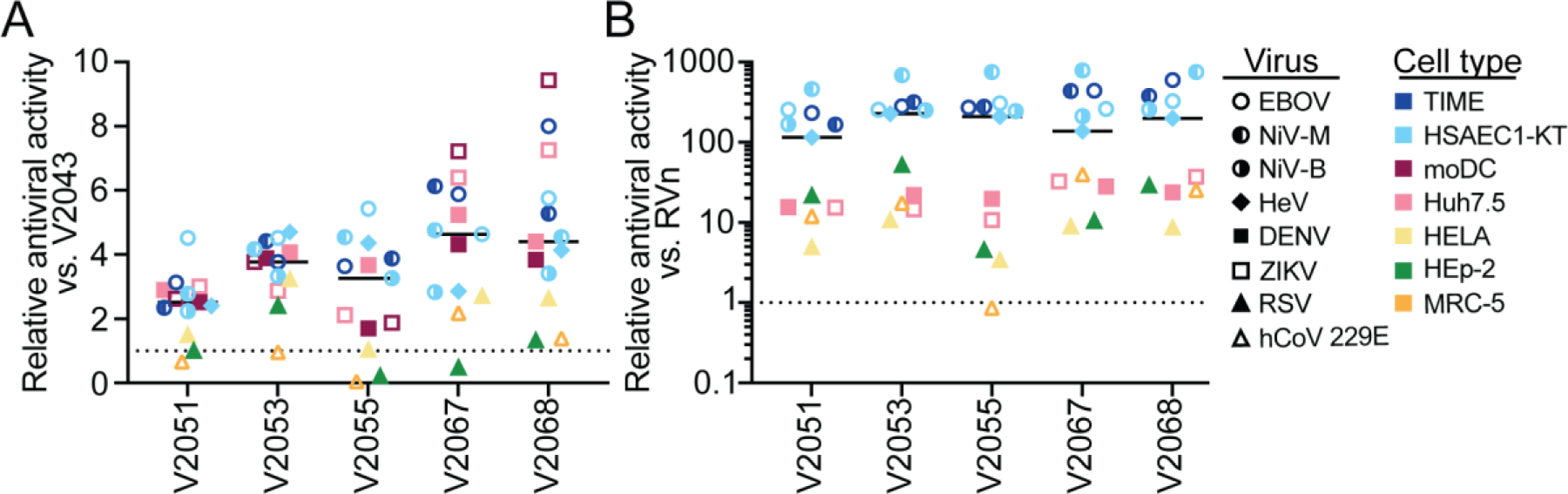
Relative antiviral activity of 3-F-4-MeO-Bn and CN R_2_ substituted compounds compared to V2043 and RVn against RNA viruses of clinical concern in several cell types. The relative average EC_50_ of lipid monophosphate prodrugs for the indicated virus and cell type compared to (A) V2043 or (B) RVn and median are shown. Average EC_50_ data are derived from at least 3 independent experiments performed in duplicate.

When comparing the efficacy of these compounds across all viruses and cell types tested, we observe that 3-F-4-MeO and CN R2 modified compounds are generally more effective than V2043 at inhibiting viral replication of DENV, ZIKV, EBOV, NiV-M, NiV-B, and HeV (median fold change relative to V2043; V2051 = 2.52, V2053 = 3.77, V2055 = 3.26, V2067 = 4.63, V2068 = 4.40) (Fig 6A). Compared to RVn, all 3-F-4-MeO and -CN R2 modified compounds tested have improved antiviral efficacy, with the exception of V2055 in RSV-infected MRC-5 cells (median fold change relative to RVn; V2051 = 115.2, V2053 = 225.9, V2055 = 209.5, V2067 = 137.1, V2068 = 198.6) (Fig 6B). Overall, this data demonstrates that 3-F-4-MeO-Bn or CN-Bn R2 modified analogs have increased antiviral activity compared to V2043 and are substantially more active than RVn against multiple RNA viral families in various cell types.

## 4. Discussion

Our lipid RVn prodrugs, including V2043, have broad spectrum activity against many RNA viruses of clinical concern in vitro and SARS-CoV-2 in vivo. Further they overcome many limitations of RDV, including requirement for IV administration and plasma instability (Schooley et al. 2021). Here, we demonstrate that modifying the R2 benzyl of V2043 to contain 3-F-4-MeO, 3-CN, or 4-CN substitutions significantly improves the antiviral activity against DENV and ZIKV in Huh7.5 cells and moDCs without significantly modifying cytotoxicity. Compounds with sn-2 benzyl 3-F-4-MeO, 3-CN, and 4-CN modifications were also efficacious at inhibiting the replication of RNA viruses in the *Filoviridae*, *Pneumoviridae*, *Paramyxoviridae*, and *Coronaviridae* families. Collectively, 3-F-4-MeO and -CN R2 modified compounds generally improve broad-spectrum antiviral activity relative to V2043.

Several oral RDV prodrugs GS-5245 (ATV006, Obeldesivir), GS-621763, and VV116 are in various stages of clinical development for the treatment of SARS-CoV-2 (Cao et al. 2022, Cao et al. 2023, Cox et al. 2021, Martinez et al. 2023 [preprint], Schäfer et al. 2022). GS-5245 and GS-621763 are rapidly metabolized to RVn (GS-441524) prior to cellular uptake in tissues, including the lung. After entry into cells, GS-5245, GS-621763, and VV116 require an initial phosphorylation step, which for nucleosides is rate-limiting (slow) and believed to account for the reduced antiviral activity of RVn compared to RDV (Eastman et al. 2020). In contrast, our oral lipid prodrugs are absorbed and circulate in plasma intact (Schooley et al. 2021). For example, Syrian hamsters administered oral V2043 maintained plasma ODBG-P-RVn levels significantly above the EC_90_ of SARS-CoV-2 12 hours post-administration with minimal conversion to RVn (Schooley et al. 2021). Additionally, V2043 and its analogues are metabolized intracellularly to RVn-monophosphate, thereby bypassing the rate-limiting initial phosphorylation step. Given that V2043 and its related analogs containing sn-2 benzyl 3-F-4-MeO, 3-CN, or 4-CN modifications are significantly more potent in vitro than RVn, we expect that these compounds will be effective oral antivirals for the treatment of many RNA viruses. Prodrug approaches which rely on extracellular RVn may be less effective against viruses which have EC_50_ values 10 to 100 times greater than V2053, 2067 and 2068 (Fig 6B) including Nipah, Ebola, Hendra, Zika and Dengue.

Our previous work has focused on the antiviral efficacy of these analogs to SARS-CoV-2 and related coronaviruses. This is the first study to investigate the broad-spectrum antiviral activity of 3-F-4-MeO, 3-CN, and 4-CN -modified V2043 analogs. Importantly, we find that these analogs have antiviral activity against viruses with pandemic potential that currently have no antiviral therapies including dengue virus, Zika virus, Ebola virus, Hendra virus and Nipah virus. A limitation of our study is that all testing was performed in vitro. However, we previously established that V2043 is effective in limiting SARS-CoV-2 infection in mice, and future studies by our labs will test the in vivo pharmacokinetics and antiviral efficacy of these compounds (Carlin et al. 2023). This work, in combination with our previous studies, provide promising evidence that V2043 and 3-F-4-MeO, 3-CN, and 4-CN modified compounds should continue development as broad-spectrum oral antivirals for the treatment of clinically significant RNA viruses.

## Supplementary Material

1

## Figures and Tables

**Table 1.** Antiviral activity of V2043 analogs to DENV and ZIKV in Huh7.5 cells

human hepatoma (Huh7.5) cell line								
Virus Family	Virus	Compound	R_1_	R_2_	EC_50_ μM	EC_90_ μM	CC_50_ μM	SI
*Flaviviridae*	DENV, UIS353	RDV	na	na	0.048 ± 0.024	0.36 ± 0.15	43.15 ± 17.99	899
		RVn	na	na	5.91 ± 2.64	17.12 ± 3.27	>100	>17
		V2043	octadecyl	(R)-Bn	1.10 ± 0.27	2.53 ± 1.12	55.79 ± 7.55	51
		V2051	octadecyl	3-F-4-MeO-Bn	0.38 ± 0.03	1.34 ± 0.91	70.13 ± 10.16	185
		V2052	oleyl	(R)-Bn	0.90 ± 0.02	3.70 ± 1.56	>100	>111
		V2053	oleyl	3-F-4-MeO-Bn	0.27 ± 0.03	1.15 ± 0.39	>100	>370
		V2054	hexadecyl	(R)-Bn	1.34 ± 0.40	5.55 ± 1.24	>100	>75
		V2055	hexadecyl	3-F-4-MeO-Bn	0.30 ± 0.16	1.30 ± 0.19	59.02 ± 3.58	197
		V2067	octadecyl	4-CN-Bn	0.21 ± 0.10	1.11 ± 0.51	47.17 ± 1.20	225
		V2068	octadecyl	3-CN-Bn	0.25 ± 0.11	1.12 ± 0.85	53.50 ± 4.86	214
	ZIKV, PRVABC59	RDV	na	na	0.27 ± 0.10	0.42 ± 0.09	43.15 ± 17.99	160
		RVn	na	na	27.31 ± 0.84	31.85 ± 2.25	>100	>4
		V2043	octadecyl	(R)-Bn	5.37 ± 1.58	11.46 ± 2.02	55.79 ± 7.55	10
		V2051	octadecyl	3-F-4-MeO-Bn	1.79 ± 0.21	3.75 ± 0.76	70.13 ± 10.16	39
		V2052	oleyl	(R)-Bn	8.53 ± 1.77	22.91 ± 1.83	>100	>12
		V2053	oleyl	3-F-4-MeO-Bn	1.87 ± 0.27	4.84 ± 1.71	>100	>53
		V2054	hexadecyl	(R)-Bn	12.51 ± 5.27	46.65 ± 10.59	>100	>8
		V2055	hexadecyl	3-F-4-MeO-Bn	2.54 ± 1.536	12.00 ± 5.23	59.02 ± 3.58	23
		V2067	octadecyl	4-CN-Bn	0.84 ± 0.17	3.37 ± 1.63	47.17 ± 1.20	56
		V2068	octadecyl	3-CN-Bn	0.74 ± 0.34	5.68 ± 2.74	53.50 ± 4.86	80

SI, selective index = CC50/EC50; Mean values with ± standard deviation values were derived from a minimum of 3 independent experiments performed in biological duplicate or triplicate. EC_50_, EC_90_, and CC_50_ values were calculated using Graphpad Prism 9 software.

**Table 2.** Antiviral activity of V2043 analogs to DENV and ZIKV in moDCs

Monocyte-derived dendritic cells (moDCs)								
Virus Family	Virus	Compound	R_1_	R_2_	EC_50_ μM	EC_90_ μM	CC_50_ μM	SI
*Flaviviridae*	DENV, UIS353	V2043	octadecyl	(R)-Bn	3.07 ± 1.26	13.85 ± 1.66	47.40 ± 14.76	15.4
		V2051	octadecyl	3-F-4-MeO-Bn	1.22 ± 0.46	6.75 ± 2.06	75.53 ± 35.68	61.9
		V2052	oleyl	(R)-Bn	2.56 ± 1.31	16.00 ± 1.37	>100	>39
		V2053	oleyl	3-F-4-MeO-Bn	0.79 ± 0.35	3.92 ± 1.31	>100	>127
		V2054	hexadecyl	(R)-Bn	6.31 ± 3.42	35.60 ± 17.38	>100	>16
		V2055	hexadecyl	3-F-4-MeO-Bn	1.80 ± 0.83	9.33 ± 1.95	>100	>56
		V2067	octadecyl	4-CN-Bn	0.71 ± 0.23	3.98 ± 1.31	29.75 ± 6.76	41.9
		V2068	octadecyl	3-CN-Bn	0.80 ± 0.22	4.00 ± 1.27	22.51 ± 9.34	28.1
	ZIKV, PRVABC59	V2043	octadecyl	(R)-Bn	7.73 ± 1.97	26.00 ± 8.39	47.40 ± 14.76	6.13
		V2051	octadecyl	3-F-4-MeO-Bn	2.95 ± 0.77	13.04 ± 5.15	75.53 ± 35.68	25.6
		V2052	oleyl	(R)-Bn	9.08 ± 2.03	55.64 ± 14.44	>100	>11
		V2053	oleyl	3-F-4-MeO-Bn	2.05 ± 0.62	9.62 ± 4.24	>100	>49
		V2054	hexadecyl	(R)-Bn	11.44 ± 4.95	45.92 ± 8.15	>100	>9
		V2055	hexadecyl	3-F-4-MeO-Bn	4.11 ± 1.64	19.80 ± 1.21	>100	>24
		V2067	octadecyl	4-CN-Bn	1.07 ± 0.44	2.79 ± 0.72	29.75 ± 6.76	27.8
		V2068	octadecyl	3-CN-Bn	0.82 ± 0.14	2.74 ± 0.88	22.51 ± 9.34	27.5

Mean values with ± standard deviation values were derived from a minimum of 3 independent experiments with moDCs derived from different donors and performed in biological duplicate. EC_50_, EC_90_, and CC_50_ values were calculated using Graphpad Prism 9 software.

**Table 3.** Antiviral activity of V2043 analogs to filoviruses and paramyxoviruses in HSAEC1-KT cells

hTERT-immortalized small airway epithelial cell line (HSAEC1-KT)								
Virus Family	Virus	Compound	R_1_	R_2_	EC_50_ μM	EC_90_ μM	CC_50_ μM	SI
*Filoviridae*	EBOV, Rec. Makona-ZsG	RDV	na	na	0.12 ± 0.06	0.58 ± 0.8	>50	>416
		RVn *	na	na	10.7 ± 2.62	21.79 ± 3.16	>100	>9.3
		V2043	octadecyl	(R)-Bn	0.19 ± 0.1	0.44 ± 0.3	10.5 ± 5.0	55
		V2051	octadecyl	3-F-4-MeO-Bn	0.042 ± 0.02	0.15 ± 0.09	10.7 ± 2.6	255
		V2053	oleyl	3-F-4-MeO-Bn	0.042 ± 0.02	0.089 ± 0.02	17.8 ± 3.4	424
		V2055	hexadecyl	3-F-4-MeO-Bn	0.035 ± 0.02	0.073 ± 0.01	18.1 ± 2.8	517
		V2067	octadecyl	4-CN-Bn	0.041 ± 0.02	0.13 ± 0.06	9.2 ± 2.1	224
		V2068	octadecyl	3-CN-Bn	0.033 ± 0.01	0.09 ± 0.06	8.9 ± 0.4	270
*Paramyxoviridae*	NiV-M, Rec. Malaysia-ZsG	RDV	na	na	0.15 ± 0.09	0.57 ± 0.6	>50	>333
		RVn *	na	na	16.46 ± 0.04	19.12 ± 0.05	>100	>6.1
		V2043	octadecyl	(R)-Bn	0.10 ± 0.06	0.32 ± 0.2	10.5 ± 5.0	105
		V2051	octadecyl	3-F-4-MeO-Bn	0.036 ± 0.02	0.13 ± 0.1	10.7 ± 2.6	297
		V2053	oleyl	3-F-4-MeO-Bn	0.024 ± 0.02	0.070 ± 0.02	17.8 ± 3.4	742
		V2055	hexadecyl	3-F-4-MeO-Bn	0.022 ± 0.01	0.087 ± 0.04	18.1 ± 2.8	823
		V2067	octadecyl	4-CN-Bn	0.021 ± 0.009	0.12 ± 0.09	9.2 ± 2.1	438
		V2068	octadecyl	3-CN-Bn	0.022 ± 0.005	0.059 ± 0.04	8.9 ± 0.4	405
		RDV	na	na	0.19 ± 0.1	1.54 ± 1.2	>50	>263
		RVn *	na	na	11.23 ± 0.63	33.6 ± 1.58	>100	>8.9
		V2043	octadecyl	(R)-Bn	0.15 ± 0.09	2.0 ± 2.4	10.5 ± 5.0	70
		V2051	octadecyl	3-F-4-MeO-Bn	0.067 ± 0.03	0.55 ± 0.3	10.7 ± 2.6	160
	NiV-B, Bangladesh	V2053	oleyl	3-F-4-MeO-Bn	0.045 ± 0.02	0.16 ± 0.06	17.8 ± 3.4	396
		V2055	hexadecyl	3-F-4-MeO-Bn	0.046 ± 0.02	0.17 ± 0.06	18.1 ± 2.8	393
		V2067	octadecyl	4-CN-Bn	0.053 ± 0.02	0.36 ± 0.09	9.2 ± 2.1	174
		V2068	octadecyl	3-CN-Bn	0.044 ± 0.006	0.15 ± 0.05	8.9 ± 0.4	202
	HeV, 1994	RDV	na	na	0.31 ± 0.2	1.21 ± 1.0	>50	>161
		RVn *	na	na	11.52 ± 1.49	26.11 ± 4.44	>100	>8.7
		V2043	octadecyl	(R)-Bn	0.24 ± 0.1	1.1 ± 1.0	10.5 ± 5.0	44
		V2051	octadecyl	3-F-4-MeO-Bn	0.10 ± 0.08	0.33 ± 0.3	10.7 ± 2.6	107
		V2053	oleyl	3-F-4-MeO-Bn	0.051 ± 0.04	0.14 ± 0.09	17.8 ± 3.4	349
		V2055	hexadecyl	3-F-4-MeO-Bn	0.055 ± 0.03	0.14 ± 0.09	18.1 ± 2.8	329
		V2067	octadecyl	4-CN-Bn	0.084 ± 0.03	0.20 ± 0.07	9.2 ± 2.1	110
		V2068	octadecyl	3-CN-Bn	0.058 ± 0.01	0.092 ± 0.03	8.9 ± 0.4	153

Mean values with ± standard deviation values were derived from a minimum of at least 3 independent experiments performed in biological triplicates. EC_50_, EC_90_, and CC_50_ values were calculated using Graphpad Prism 9 software.

* RVn values are from Lo et al. Microbiol Spectr 2021 (PMID 34817209).

**Table 4.** Antiviral activity of V2043 analogs to EBOV and NiV in TIME cells

Primary-like human telomerase reverse transcriptase (hTERT)-immortalized human microvascular endothelial (TIME)								
Virus Family	Virus	Compound	R_1_	R_2_	EC_50_ μM	EC_90_ μM	CC_50_ μM	SI
*Filoviridae*	EBOV, Rec. Makona- ZsG	RDV	na	na	0.035 ± 0.01	0.16 ± 0.08	14.7 ± 1.8	420
		RVn *	na	na	14.88 ± 0.28	17.24± 0.16	>100	>3.36
		V2043	octadecyl	(R)-Bn	0.20 ± 0.1	0.48 ± 0.2	31.3 ± 8.1	157
		V2051	octadecyl	3-F-4-MeO-Bn	0.064 ± 0.03	0.14 ± 0.08	36.8 ± 7.8	575
		V2053	oleyl	3-F-4-MeO-Bn	0.053 ± 0.01	0.13 ± 0.05	>50	>943
		V2055	hexadecyl	3-F-4-MeO-Bn	0.055 ± 0.02	0.13 ± 0.05	48.2 ± 6.6	876
		V2067	octadecyl	4-CN-Bn	0.034 ± 0.01	0.20 ± 0.2	27.5 ± 7.7	809
		V2068	octadecyl	3-CN-Bn	0.025 ± 0.005	0.10 ± 0.06	37.2 ± 2.3	1488
*Paramyxoviridae*	NiV-M, Rec. Malaysia- ZsG	RDV	na	na	0.041 ± 0.02	0.13 ± 0.08	14.7 ± 1.8	359
		RVn *	na	na	13.53 ± 2.44	17.52± 0.77	>100	>3.7
		V2043	octadecyl	(R)-Bn	0.19 ± 0.1	0.30 ± 0.1	31.3 ± 8.1	165
		V2051	octadecyl	3-F-4-MeO-Bn	0.081 ± 0.06	0.14 ± 0.1	36.8 ± 7.8	454
		V2053	oleyl	3-F-4-MeO-Bn	0.043 ± 0.01	0.08 ± 0.05	>50	>1162
		V2055	hexadecyl	3-F-4-MeO-Bn	0.049 ± 0.01	0.10 ± 0.06	48.2 ± 6.6	984
		V2067	octadecyl	4-CN-Bn	0.031 ± 0.004	0.24 ± 0.2	27.5 ± 7.7	887
		V2068	octadecyl	3-CN-Bn	0.036 ± 0.02	0.11 ± 0.09	37.2 ± 2.3	1033

Mean values with ± standard deviation values were derived from a minimum of at least 3 independent experiments performed in biological triplicates. EC_50_, EC_90_, and CC_50_ values were calculated using Graphpad Prism 9 software.

* RVn values are from Lo et al. Microbiol Spectr 2021 (PMID 34817209).

**Table 5.** Antiviral activity of V2043 analogs to RSV and HCoV in human cells

Virus Family	Virus	Cell type	Compound	R_1_	R_2_	EC_50_ μM	EC_90_ μM	CC_50_ μM	SI
*Pneumoviridae*	RSV	HeLa	RDV	na	na	0.08 ± 0.06	0.25 ± 0.2	26.4 ± 3.8	330
			RVn	na	na	0.33 ± 0.1	1.93 ± 1.5	>50	151
			V2043	octadecyl	(R)-Bn	0.10 ± 0.03	0.31 ± 0.2	34.9 ± 17.1	349
			V2051	octadecyl	3-F-4-MeO-Bn	0.067 ± 0.03	0.16 ± 0.1	39.1 ± 13.7	584
			V2053	oleyl	3-F-4-MeO-Bn	0.031 ± 0.01	0.11 ± 0.08	46.1 ± 8.6	1487
			V2055	hexadecyl	3-F-4-MeO-Bn	0.097 ± 0.03	0.24 ± 0.15	50.6 ±.8	522
			V2067	octadecyl	4-CN-Bn	0.037 ± 0.033	0.089 ± 0.07	5.1 ± 3.4	138
			V2068	octadecyl	3-CN-Bn	0.038 ± 0.00	0.170 ± 0.08	34.7 ± 15.1	913
*Pneumoviridae*	RSV	HEp-2	RDV	na	na	0.003 ± 0.001	0.005 ± 0.004	8.1 ± 1.3	2700
			RVn	na	na	0.26 ± 0.05	0.63 ± 1.7	>50	>192
			V2043	octadecyl	(R)-Bn	0.012 ± 0.001	0.023 ± 0.004	30.8 ± 11.7	2567
			V2051	octadecyl	3-F-4- MeO-Bn	0.012 ± 0.001	0.034 ± 0.009	24.7 ± 10.9	2058
			V2053	oleyl	3-F-4-MeO-Bn	0.005 ± 0.003	0.012 ± 0.007	37.6 ± 15.0	7520
			V2055	hexadecyl	3-F-4-MeO-Bn	0.057 ± 0.03	0.90 ± 1.4	28.1 ± 2.4	493
			V2067	octadecyl	4-CN-Bn	0.025 ± 0.01	0.088 ± 0.04	5.9 ± 1.2	236
			V2068	octadecyl	3-CN-Bn	0.009 ± 0.003	0.020 ± 0.01	15.1 ± 2.0	1678
*Coronaviridae*	HCo V 229E	MRC-5	RDV	na	na	0.08 ± 0.03	0.87 ± 0.5	>50	>625
			RVn	na	na	1.31 ± 0.8	4.10 ± 4.6	>50	>38
			V2043	octadecyl	(R)-Bn	0.072 ± 0.03	0.12 ± 0.07	>50	>694
			V2051	octadecyl	3-F-4-MeO-Bn	0.109 ± 0.005	0.31 ± 0.1	>50	>458
			V2052	oleyl	(R)-Bn	0.164 ± 0.02	0.50 ± 0.4	>50	>304
			V2053	oleyl	3-F-4-MeO-Bn	0.075 ± 0.01	0.20 ± 0.04	>50	>666
			V2054	hexadecyl	(R)-Bn	0.87 ± 0.7	4.24 ± 4.8	>50	>57
			V2055	hexadecyl	3-F-4-MeO-Bn	1.52 ± 0.1	30.6 ± 15.8	>50	>32
			V2067	octadecyl	4-CN-Bn	0.033 ± 0.02	0.047 ± 0.02	>50	>1515
			V2068	octadecyl	3-CN-Bn	0.052 ± 0.04	0.092 ± 0.08	38.3	737

Mean values with ± standard deviation values were derived from a minimum of at least 2 independent experiments performed in biological triplicates. EC_50_, EC_90_, and CC_50_ values were calculated using Graphpad Prism 9 software.

## Highlights:

- Addition of 3-F-4-MeO, 3-CN, or 4-CN to the 2-O-benzyl-sn-glyceryl ester increases in vitro antiviral potency of lipid-modified monophosphate prodrugs of remdesivir nucleoside compared to V2043
- 3-F-4-MeO, 3-CN, and 4-CN derivatives are active in many cell types, including against flaviviruses in primary human dendritic cells
  3-F-4-MeO, 3-CN, and 4-CN derivatives are broadly active against clinically significant RNA viruses from *Flaviviridae*, *Filoviridae*, *Paramyxoviridae, Pneumoviridae,* and *Coronaviridae* families

## Highlights:

- Lipid RVn monophosphate prodrugs are potent broad spectrum oral antivirals
- 3-F-4-MeO, 3-CN, or 4-CN modifications increase in vitro antiviral potency
- 3-F-4-MeO, 3-CN, or 4-CN modified prodrugs are active in many cell types
- These prodrugs are potent inhibitors of RNA viruses with pandemic potential
