# Supplementary material for: Enhanced Broad Spectrum In Vitro Antiviral Efficacy of 3-F-4-MeO-Bn, 3-CN, and 4-CN Derivatives of Lipid Remdesivir Nucleoside Monophosphate Prodrugs
Source: Antiviral Res. Author manuscript; Available in PMC 2024 Feb 5. (PMC10790242; doi:10.1016/j.antiviral.2023.105718)
Supplement: 1 [file NIHMS1936038-supplement-1.docx]

Title: Enhanced Broad Spectrum In Vitro Antiviral Efficacy of 3-F-4-MeO-Bn, 3-CN, and 4-CN Derivatives of Lipid Remdesivir Nucleoside Monophosphate Prodrugs

Authors: Rachel E. McMillan^1,2,#^, Michael K. Lo^3,#^, Xing-Quan Zhang^1^, James R. Beadle^1^, Nadejda Valiaeva^1^, Aaron F. Garretson^1,2^, Alex E. Clark^1,2^, Jon E. Freshman^1,2^, Joyce Murphy^1^, Joel M. Montgomery^3^, Christina F. Spiropoulou^3^, Robert T. Schooley^1^, Karl Y. Hostetler^1^, Aaron F. Carlin^1,2^*

^1^Division of Infectious Diseases and Global Public Health, Department of Medicine, University of California San Diego, School of Medicine, La Jolla, California, USA.

^2^Department of Pathology, University of California San Diego, School of Medicine, La Jolla, California, USA.

^3^Viral Special Pathogens Branch, Centers for Disease Control and Prevention, Department of Health and Human Services, Atlanta, Georgia, USA.

^#^Authors contributed equally

Corresponding author: Aaron F. Carlin, acarlin@health.ucsd.edu


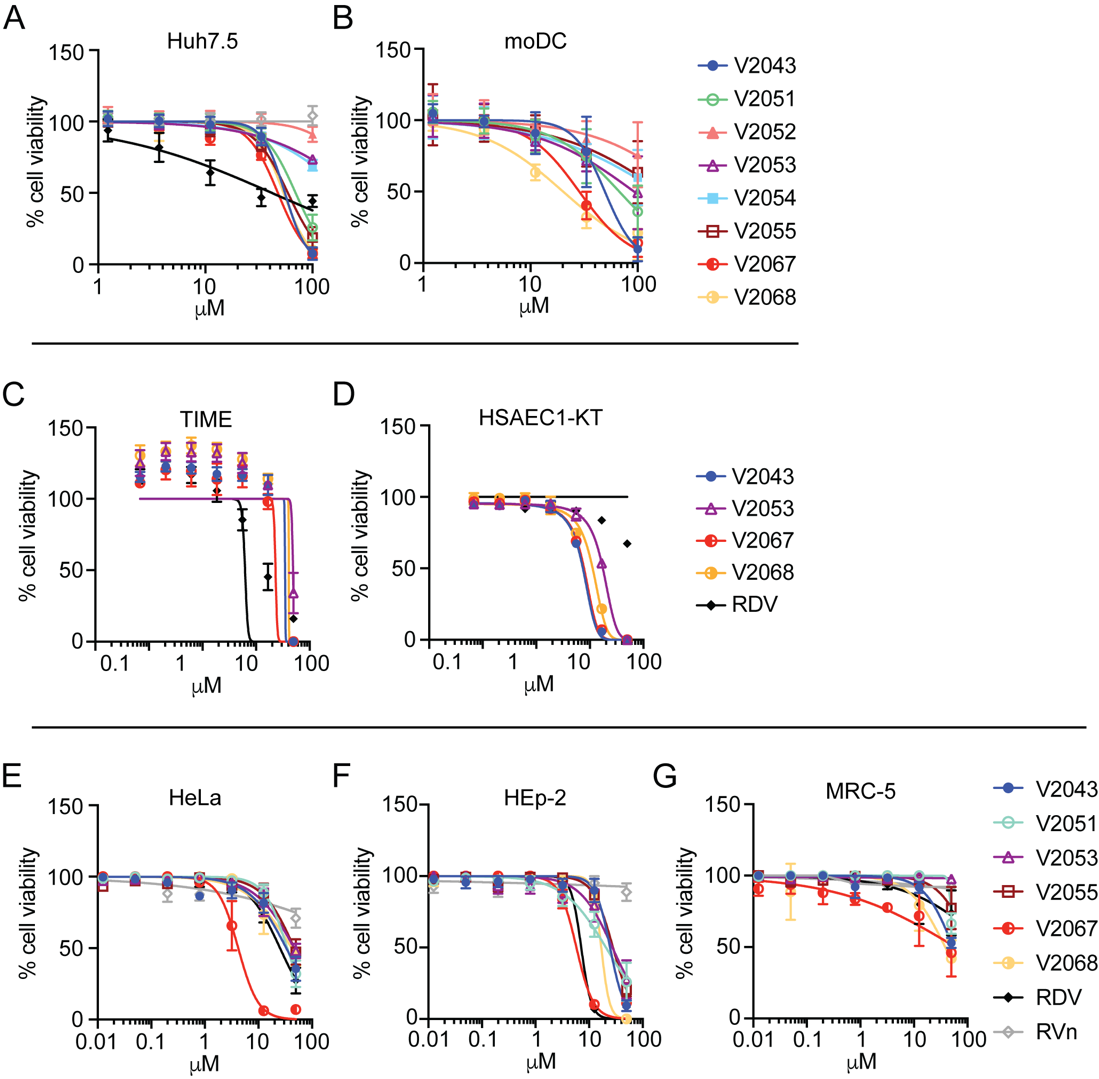


**Supplementary Figure 1: Antiviral cytotoxicity in multiple cell types.** (A-B) Cytotoxicity dose-response curves to V2043 and V2051-V2055 in Huh7.5 and moDCs. (B-G) Cytotoxicity dose-response curves to RDV, V2043, and V2051-V2055 in TIME, HSAEC1-KT, HEp-2, HeLa, and MRC-5 cells. Data shown are generated from at least 3 independent experiments performed in duplicate. Error bars represent standard deviation.
