# Supplementary material for: Enhanced broad spectrum in vitro antiviral efficacy of 3-F-4-MeO-Bn, 3-CN, and 4-CN derivatives of lipid remdesivir nucleoside monophosphate prodrugs
Source: Antiviral Res. 2023 Nov;219:105718. doi: 10.1016/j.antiviral.2023.105718 (PMC10790242; doi:10.1016/j.antiviral.2023.105718)
Supplement: Supplementary file 1 [file mmc1.docx]

**Supplementary Figure 1: Antiviral cytotoxicity in multiple cell types.** (A-B) Cytotoxicity dose-response curves to V2043 and V2051-V2055 in Huh7.5 and moDCs. (B-G) Cytotoxicity dose-response curves to RDV, V2043, and V2051-V2055 in TIME, HSAEC1-KT, HEp-2, HeLa, and MRC-5 cells. Data shown are generated from at least 3 independent experiments performed in duplicate. Error bars represent standard deviation.
